# Left Ventricular Geometrical Changes in Severely Obese Adolescents: Prevalence, Determinants, and Clinical Implications

**DOI:** 10.1007/s00246-020-02487-9

**Published:** 2020-10-20

**Authors:** Ali Talib, Yvonne G. M. Roebroek, Givan F. Paulus, Kris van Loo, Bjorn Winkens, Nicole D. Bouvy, Ernst L. W. E. van Heurn

**Affiliations:** 1 Department of Surgery, Maastricht University Medical Center, P. Debyelaan 25, 6229 HX Maastricht, The Netherlands; 2 NUTRIM School for Nutrition and Translational Research in Metabolism, Maastricht University, Maastricht, The Netherlands; 3 Department of General Surgery, Maxima Medical Center, Veldhoven, The Netherlands; 4 Department of General Surgery, Spaarne Gasthuis, Haarlem, The Netherlands; 5 Department of Pediatrics, Maastricht University Medical Center, Maastricht, The Netherlands; 6 Department of Methodology and Statistics, CAPHRI Care and Public Health Research Institute, Maastricht University, Maastricht, The Netherlands; 7 Department of Pediatric Surgery, Amsterdam University Medical Centers, Amsterdam, The Netherlands

**Keywords:** Left ventricular hypertrophy, Left ventricular geometry, Severe obesity, Adolescents

## Abstract

Left ventricular hypertrophy (LVH) is independently associated with a higher risk of cardiovascular morbidity and mortality in adults. Adiposity is a risk factor for LVH, independent of blood pressure. Potential causes of this nonhemodynamic pathogenesis identified in adults include adverse body fat distribution, insulin resistance, dyslipidemia, and obstructive sleep apnea syndrome (OSA). In severely obese adolescents, the determinants of obesity-induced changes in left ventricular structure are poorly characterized. Cardiac ultrasonographic, demographic, anthropometric, and comorbidity-related data were prospectively collected in adolescents with severe obesity refractory to conservative treatment who presented for surgical therapy. Differences between adolescents with LVH and without LVH were evaluated using independent samples *t*, chi-square, or Fisher’s exact test. Multivariable linear regression analysis was performed to evaluate associations with left ventricular structural changes, corrected for body mass index (BMI) *z* score. Forty-three patients entered analysis, of whom 24 (55.8%) showed LVH. The most common geometrical change was eccentric LVH (eLVH), occurring in 21 subjects (48.8%). Demographic and anthropometric variables did not differ between patients with and without LVH. Independent of BMI *z* score, left ventricular mass index was significantly associated with apnea–hypopnea index (AHI) (regression parameter *B* = 0.8; 95% CI 0.3 to 1.2). Interventricular septum thickness (IVST) was significantly associated with HOMA-IR values (*B* = 0.1; 95% CI 0.04 to 0.2), HDL-cholesterol (*B* = − 1.2; 95% CI − 2.2 to 0.1), and triglyceride levels (*B* = 0.5; 95% CI 0.001 to 0.9). LVH, especially eLVH, is highly prevalent amongst severely obese adolescents. Adverse changes in cardiac structure, increased IVST in particular, are independently associated with several nonhemodynamic comorbidities that are common in this population, namely OSA, insulin resistance, and dyslipidemia.

## Introduction

Pediatric obesity is associated with adverse changes in left ventricular geometry, especially left ventricular hypertrophy (LVH) [1–6]. In adults, LVH increases the risk of ventricular arrhythmias and decompensation, and poses a fourfold increased risk of cardiovascular morbidity and mortality [7]. In hypertensive patients, the presence of LVH increases the risk of cardiovascular events by a factor 20 [8].

The reported prevalence of LVH in obese children, adolescents, and young adults between the ages of 3 and 22 years old ranges from 10 to 46% [1–6]. Although LVH is normally asymptomatic in children, multiple prospective longitudinal studies have established that several risk factors for adverse cardiovascular events persist into adulthood [9, 10]. Interestingly, on long-term follow-up of patients with essential hypertension, (partial) reversal of LVH by antihypertensive treatment has been found to reduce the risk of cardiovascular morbidity and mortality to the same level as those who did not have LVH at the beginning of follow-up [11]. This provides an argument for early detection of LVH in obese individuals and treatment of causative factors.

Adiposity is a predictor of LVH in both hypertensive and nonhypertensive children, independent of age [12–15]. The mechanisms underlying obesity-induced changes in left ventricular geometry are diverse and include hemodynamic factors (hypertension, increased intravascular volume), metabolic factors, hormonal factors, and factors related to OSA [16]. In the Bogalusa Heart study, it was found that young adults (aged 24–44 years) were more likely to suffer from LVH if they were exposed to risk factors of LVH in childhood, including obesity, diabetes, increased waist circumference, elevated systolic blood pressure (SBP) and diastolic blood pressure (DBP), insulin resistance, and dyslipidemia [17].

The aim of our research is to improve understanding of the prevalence and determinants of left ventricular geometrical changes in a homogeneous and well-characterized population of severely obese adolescents. We hypothesize that LVH, especially eccentric LVH, is highly prevalent in our population, that BMI is the most important anthropometric predictor of LVH, and that LVH is independently associated with several pathogenic factors, including blood pressure and variables related to insulin sensitivity, lipid profile, hormonal aberrancies, and OSA.

## Methods

This study was conducted as part of a randomized controlled trial for adolescents eligible for bariatric surgery (BASIC trial, NCT01172899) [18]. The study population of the BASIC trial consists of severely obese adolescents who all have been treated extensively for their severe obesity by conservative methods during at least 12 months without effect. The current study was based on the baseline measurements of this trial, before randomization took place.

### Participants

All patients included in the BASIC trial who received a baseline cardiac ultrasound examination were used for this baseline analysis. Detailed information regarding the BASIC trial study design, in- and exclusion criteria, and randomization process was published previously [18]. In summary, inclusion criteria were age 14–16 years; sex- and age-adjusted BMI ≥ 40 kg/m^2^ (or ≥ 35 kg/m^2^ combined with presence of obesity-associated comorbidity); and participation in combined lifestyle interventions during at least 12 months without adequate weight loss (defined as 5% total body weight loss). In order to maintain a homogenous study population with regard to pubertal status, girls were excluded if they were premenarchal, boys if their bone age was < 15 years.

All participants were subjected to standardized comprehensive baseline measurements and investigations in order to exclude (subclinical) conditions causing obesity.

### Measurements

All measurements within one patient were carried out during a single visit. Body height and weight were measured using a stadiometer and digital scale, respectively, with patients dressed in underwear. A tape measure was used for standardized measurement of body circumferences at neck level, abdominal level, and hip level. BMI was calculated as [body weight]/[body height*body height] in kg/m^2^, and BMI *z* scores were calculated using Cole’s LMS method [19]. Daytime blood pressure was measured while the patient was resting, during a period of 60 to 90 min with intervals of 3 min between measurements, using the Mobil-O-Graph® NG (I.E.M. GmbH, Stolberg, Germany). Prehypertension and hypertension were defined according to the fourth report from the National High Blood Pressure Education Program, and blood pressure *z* scores were calculated according to the method described in that same report [20].

A fasting blood draw was performed to measure serum glucose, insulin, cholesterol, triglycerides, free fatty acids, glycated hemoglobin (HbA1C), and leptin. HOMA-IR was calculated according to the method described by Mathews et al., i.e., ((fasting insulin (microU/l)/(fasting glucose (mmol/l))/22.5, where insulin was converted from pmol/l to microU/l by dividing by 6.945 [21].

Video-assisted 12 channel polysomnography (PSG) (Brain RT, OSG, Rumst, Belgium) was performed at the pediatrics department of Maastricht University Medical Center. The scoring of sleep stages and respiratory-related events were performed by a single specialized analyst using the American Academy of Sleep Medicine (AASM) 2012 updated guidance for scoring pediatric respiratory events [22].

All cardiac ultrasounds were planned with a single pediatric cardiologist, who reported interventricular septal thickness (IVST), left ventricular end-diastolic posterior wall thickness (LVPWT), left ventricular end-diastolic dimension (LVEDD), and left ventricular end-systolic dimension (LVESD). In the absence of this pediatric cardiologist, another experienced pediatric cardiologist was consulted to perform the ultrasound examination.

Left ventricular mass (LVM) was calculated using the Devereux formula, where left ventricular mass (in grams) is equal to 0.8(1.04((LVEDD + IVST + LPWT)^3^ − LVEDD^3^)) + 0.6 [23]. Left ventricular mass was subsequently indexed by dividing it by height to the power of 2.7, which was previously described as the optimal height exponent between children and adults for indexing LVM [24, 25]. Thus, left ventricular mass index (LVMI) is equal to LVM divided by height^2.7^ (height in meters). LVH was defined as a LVMI ≥ 38.6 g/m^2.7^, in accordance with the 95th percentile of LVM in a cohort of 192 healthy children of 6 to 17 years old [25].

Cardiac geometry was further specified using the relative wall thickness (RWT) and LVMI, according to the method of Ganau et al., where RWT was calculated as 2*LVPWT/LVEDD [26]. In children, an RWT > 0.41, corresponding to the 95th percentile of RWT in children and adolescents, corresponds to concentric changes, and an RWT ≤ 0.41 corresponds to normal geometry or eccentric changes [27]. We defined normal geometry and concentric left ventricular remodeling (cLVR) as a normal LVMI (< 38.6 g/m^2.7^) with an RWT that is ≤ 0.41 or > 0.41, respectively. We defined left ventricular hypertrophy as a LVMI ≥ 38.6 g/m^2.7^ and further specified this as eccentric left ventricular hypertrophy (eLVH) if RWT was ≤ 0.41 and concentric left ventricular hypertrophy when RWT was > 0.41.

### Statistical Analysis

Numerical data are presented as mean ± standard deviation and range where appropriate. Categorical data are presented as number (percentage). Demographic and clinical variables were compared between patients with no LVH and patients with LVH, using an independent samples *t* test for numerical variables and *χ*^2^ or Fisher’s exact test for categorical variables. Multivariable linear regression analysis was used to evaluate the association between several relevant demographic, anthropometric, and comorbidity endpoints and LVMI, correcting for BMI *z* score. As sensitivity analysis, these analyses were repeated, where we additionally corrected for age and gender. The associations between the dependent and independent variables were assessed separately as the sample size was too small to include all independent variables in one regression model. All assumptions were checked using plots (scatterplots for linearity, *Q*–*Q*-plots and histograms for normality, residual plots for homoscedasticity), where Cook’s distance > 1 was used to define an influential outlier. In case normality was violated, a log or square-root transformation was considered to achieve normality. IBM SPSS Statistics for Windows (version 24.0; Armonk, NY, USA) was used for the aforementioned statistical analyses. Two-sided *p*-value ≤ 0.05 was considered statistically significant.

## Results

Of the 60 adolescents included in the BASIC trial, 43 subjects received a baseline cardiac ultrasound, of whom 42 were performed by CvL. Seventeen patients did not receive an ultrasound due to logistic circumstances, mainly unavailability of a pediatric cardiologist.

The baseline characteristics of the 43 remaining adolescents are presented in Table 1. The majority (79.0%) was female; mean (± SD) age was 15.7 ± 1.0 years; and BMI was on average 44.2 ± 5.5 kg/m^2^, with a mean BMI *z* score of 3.5 ± 0.3.

**Table 1:** Demographic and anthropometric characteristics for overall population and patients with LVH. * Fischer’s exact test performed

Variable	Overall (*n* = 43)	No LVH (*n* = 19)	LVH (*n* = 24)	*p*-value
Demographic variables				
Age (years)	15.7 (± 1.0)	15.8 (± 1.0)	15.6 (± 0.9)	0.657
Gender female (%)	34 (79.0%)	17 (89.4%)	17 (70.8%)	0.257*
Anthropometric variables				
BMI (kg/m^2^)	44.2 (± 5.5)	45.2 (± 5.5)	43.5 (± 5.5)	0.330
BMI *z* score	3.5 (± 0.3)	3.6 (± 0.3)	3.5 (± 0.3)	0.420
Neck circumference (cm)	40.9 (± 3.5)	41.4 (± 3.2)	40.6 (± 3.7)	0.423
Abdominal circumference (cm)	126.5 (± 13.7)	127.8 (± 15.6)	125.6 (± 12.4)	0.625
Hip circumference (cm)	131.7 (± 10.3)	132.5 (± 9.2)	131.2 (± 11.2)	0.674
Waist-length ratio	0.7 (± 0.2)	0.7 (± 0.3)	0.7 (± 0.1)	0.466
Waist-hip ratio	1.0 (± 0.9)	1.0 (± 0.1)	1.0 (± 0.1)	0.835
Fat free mass (%)	66.2 (± 11.1)	69.6 (± 15.0)	66.1 (± 7.6)	0.336

**Fig. 1:**
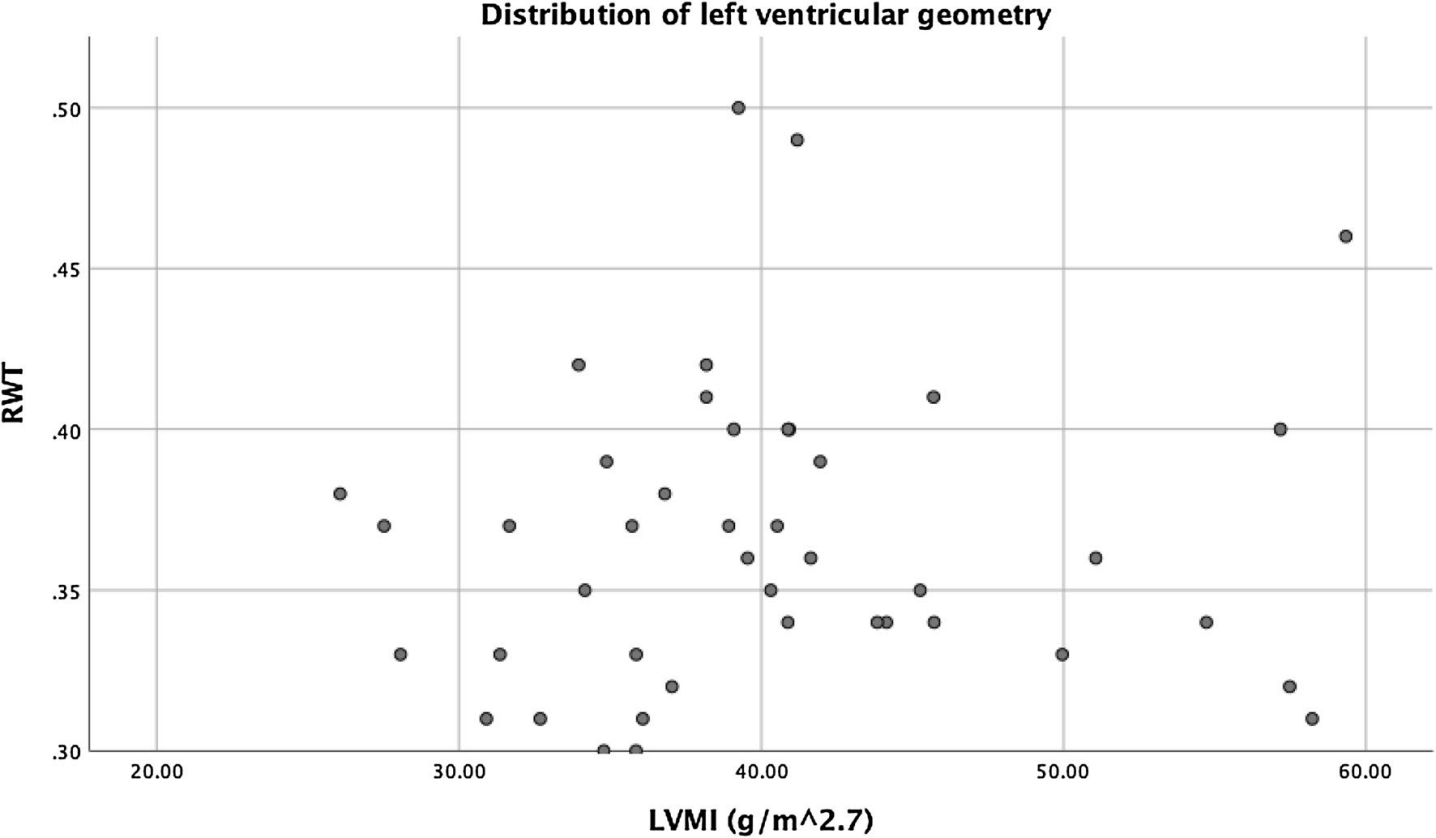
Distribution of left ventricular geometry in morbidly obese adolescents. *RWT* relative wall thickness, *LVMI* left ventricular mass index. A LVMI of > 38.6 g/m^2.7^ was considered as hypertrophy. A RWT > 0.41 was regarded as concentric LVH or concentric remodeling, depending on the LVMI. A RWT ≤ 0.41 was regarded as eccentric LVH or normal geometry, depending on the LVMI

The mean values measured with cardiac ultrasonography in our study population were: LVM of 170.4 g (± 42.3), LVMI of 40.4 g/m^2.7^ (± 8.3), IVST of 9.6 mm (± 1.3), LVPWT of 9.1 mm (± 1.0), and LVEDD of 50.3 mm (± 4.7). The average RWT was 0.37 (± 0.05). Twenty-four patients (55.8%) had a LVMI of ≥ 38.6 g/m^2.7^, corresponding to LVH according to the pediatric definition [25]. Figure 1 shows the geometrical distribution in our study population. eLVH, cLVH, and cLVR occurred in 21 (48.8%), three (7.0%), and two (4.7%) patients, respectively.

Differences in demographic, anthropometric, and potentially pathogenic factors between patients with LVH and without LVH are shown in Tables 1 and 2. We did not observe any statistically significant differences in these factors between patients with LVH and patients without LVH. As a sensitivity analysis, we further analyzed our data using the adult cut-off for LVH (≥ 51.0 g/m^2.7^), upon which only six patients (14.0%) would have LVH with no statistically significant differences between the group with LVH and without LVH.

**Table 2:** Potentially pathogenic characteristics for overall population and patients with LVH

Variable	Overall (*n* = 43)	No LVH (*n* = 19)	LVH (*n* = 24)	*p*-value
Blood pressure related				
SBP (mmHg)	121.4 (± 11.3)	118.7 (± 11.4)	123.3 (± 11.0)	0.221
DBP (mmHg)	69.5 (± 9.1)	68.0 (± 9.5)	70.5 (± 9.0)	0.417
*z* score SBP	0.7 (± 1.0)	0.4 (± 1.1)	0.9 (± 1.0)	0.134
*z* score DBP	0.3 (± 0.8)	0.2 (± 0.9)	0.4 (± 0.7)	0.485
Glycemic control				
Fasting glucose (mmol/l)	5.4 (± 1.8)	5.6 (± 2.7)	5.2 (± 0.5)	0.451
Fasting insulin (pmol/l)	208.1 (± 126.0)	210.6 (± 144.0)	207.2 (± 113.8)	0.933
HbA1c (%)	5.5 (± 1.3)	5.6 (± 2.0)	5.4 (± 0.3)	0.663
HOMA-IR	7.3 (± 4.9	7.6 (± 5.5)	7.1 (± 4.5)	0.705
Lipid profile				
TC (mmol/l)	4.7 (± 0.9)	4.7 (± 0.9)	4.6 (± 0.9)	0.705
HDL-c (mmol/l)	1.1 (± 0.4)	1.1 (± 0.5)	1.1 (± 0.3)	0.477
Ratio TC/HDL-c	4.7 (± 1.7)	4.7 (± 2.1)	4.6 (± 1.4)	0.801
LDL-c (mmol/l)	2.7 (+ 0.9)	2.8 (± 1.0)	2.7 (± 0.8)	0.590
FFA (mmol/l)	0.8 (± 0.3)	0.8 (± 0.3)	0.9 (± 0.2)	0.599
Triglycerides (mmol/l)	1.9 (± 0.9)	1.8 (± 1.0)	1.9 (± 0.8)	0.855
OSA-related variables				
AHI	5.8 (± 5.2)	4.3 (± 3.6)	7.0 (± 6.1)	0.119
Average O_2_-dip (%)	3.0 (± 0.5)	3.0 (0.7)	2.9 (0.3)	0.385

By contrast, multivariable linear regression analysis, correcting for BMI *z* score, showed that increased LVMI was associated with higher AHI (see Table 3). In our study population, 38 patients received polysomnographic evaluation (88.4%). The mean AHI was 5.8 (± 5.2), ranging from 0 to 20.1. For purpose of this analysis, we square-root transformed AHI, which had a right-skewed distribution, to sqrtAHI, upon which normal distribution was achieved. We found that higher LVMI was associated with higher AHI (regression coefficient *B* = 0.8; 95% CI 0.3 to 1.2) as well as sqrtAHI (*B* = 3.6; 95% CI 1.3 to 5.9) after correction for BMI *z* score. The association of AHI and sqrtAHI with LVMI persisted after further correcting for age and sex in addition to BMI *z* score (*B* = 0.8; 95% CI 0.3 to 1.3, and *B* = 3.7; 95% CI 1.3 to 6.1).

**Table 3:** Unstandardized regression coefficients B and 95% confidence intervals, indicating associations of various variables with left ventricular geometrical indices, corrected for BMI *z* score. Statistically significant differences emphasized with a bold font

	LVMI (g/m^2.7^)	LVM (g)	IVST (mm)	LPWT (mm)	LVEDD (mm)
Demographic variables					
Age (years)	0.6 (− 2.2 to 3.4)	4.6 (− 9.4 to 18.6)	0.06 (− 0.3 to 0.5)	− 0.1 (− 0.5 to 0.2)	0.9 (− 0.6 to 2.5)
Gender female	− 4.3 (− 11.0 to 2.4)	**− 68.1 (− 95.3 to 40.9)**	**− 1.9 (− 2.7 to 1.1)**	− 0.4 (− 1.2 to 0.4)	**− 6.6 (− 9.8 to 3.3)**
Anthropometric variables					
BMI (kg/m^2^)*	− 0.1 (− 0.6 to 0.3)	− 0.8 (− 3.3 to 1.6)	− 0.006 (− 0.08 to 0.07)	− 0.004 (− 0.06 to 0.05)	− 0.1 (− 0.4 to 0.1)
BMI *z* score*	− 1.5 (− 9.9 to 6.9)	5.7 (− 37.3 to 48.7)	0.2 (− 1.0 to 1.5)	− 0.2 (− 0.8 to 1.2)	− 0.3 (− 5.2 to 4.5)
Neck circumference (cm)	− 0.5 (− 1.5 to 0.5)	3.2 (− 1.7 to 8.1)	0.1 (− 0.006 to 0.3)	− 0.02 (− 0.1 to 0.1)	0.2 (− 0.4 to 0.7)
Abdominal circumference (cm)	0.01 (− 0.3 to 0.3)	1.0 (− 0.5 to 2.5)	0.03 (− 0.02 to 0.07)	− 0.002 (− 0.04 to 0.03)	0.1 (− 0.03 to 0.3)
Hip circumference (cm)	− 0.1 (− 0.6 to 0.3)	− 0.9 (− 3.0 to 1.1)	− 0.006 (− 0.07 to 0.06)	0.01 (− 0.04 to 0.06)	− 0.2 (− 0.4 to 0.07)
Waist-length ratio	4.1 (− 9.0 to 17.2)	− 5.7 (− 73.0 to 61.6)	0.2 (− 1.8 to 2.1)	− 0.6 (− 1.0 to 2.1)	− 2.6 (− 10.1 to 4.8)
Waist-hip ratio	9.741 (− 23.2 to 42.7)	144.2 (− 18.4 to 306.9)	3.2 (− 1.6 to 8.1)	− 0.8 (− 4.7 to 3.2)	**21.5 (3.9 to 39.1)**
Fat free mass (%)	− 0.2 (− 0.4 to 0.1)	− 0.6 (− 1.8 to 0.7)	− 0.01 (− 0.05 to 0.02)	− 0.005 (− 0.04 to 0.02)	− 0.07 (− 0.2 to 0.07)
Blood pressure related					
SBP (mmHg)	0.2 (− 0.1 to 0.4)	0.9 (− 0.3 to 2.2)	0.03 (− 0.006 to 0.07)	0.02 (− 0.01 to 0.04)	0.07 (− 0.8 to 0.2)
DBP (mmHg)	0.1 (− 0.2 to 0.4)	0.6 (− 0.9 to 2.1)	0.03 (− 0.02 to 0.08)	0.02 (− 0.02 to 0.05)	− 0.002 (− 0.2 to 0.2)
* z* score SBP	1.7 (− 0.8 to 4.2)	0.1 (− 13.2 to 13.4)	0.1 (− 0.3 to 0.5)	0.1 (− 0.2 to 0.4)	− 0.3 (− 1.8 to 1.1)
*z* score DBP	1.9 (− 1.5 to 5.2)	0.2 (− 17.0 to 17.4)	0.2 (− 0.3 to 0.7)	0.2 (− 0.2 to 0.6)	− 0.8 (− 2.7 to 1.1)
Glycemic control					
Fasting glucose (mmol/l)	− 1.2 (− 2.6 to 0.2)	− 3.1 (− 10.4 to 4.3)	− 0.01 (− 0.2 to 0.2)	− 0.05 (− 0.2 to 0.1)	− 0.5 (− 1.3 to 0.3)
Fasting insulin (pmol/l)	− 0.001 (− 0.02 to 0.02)	0.04 (− 0.07 to 0.2)	**0.005 (0.002 to 0.007)**	0.001 (− 0.002 to 0.003)	− 0.002 (− 0.02 to 0.01)
HbA1c (%)	− 1.7 (− 3.6 to 0.3)	− 5.5 (− 15.6 to 4.6)	− 0.03 (− 0.3 to 0.3)	− 0.08 (− 0.3 to 0.2)	− 0.8 (− 1.9 to 0.3)
HOMA-IR	− 0.2 (− 0.8 to 0.4)	0.6 (− 2.3 to 3.6)	**0.1 (0.04 to 0.2)**	0.005 (− 0.06 to 0.07)	− 0.1 (− 0.5 to 0.2)
Lipid profile					
TC (mmol/l)	− 1.6 (− 4.6 to 1.3)	− 6.0 (− 21.2 to 9.1)	0.01 (− 0.4 to 0.5)	− 0.05 (− 0.4 to 0.3)	− 1.2 (− 2.8 to 0.5)
HDL-c (mmol/l)	− 4.8 (− 12.3 to 2.6)	− 32.8 (− 70.3 to 4.7)	**− 1.2 (− 2.2 to 0.1)**	− 0.7 (− 1.5 to 0.2)	− 1.6 (− 5.9 to 2.7)
Ratio TC/HDL-c	0.1 (− 1.5 to 1.7)	2.4 (− 5.6 to 10.4)	0.2 (− 0.07 to 0.4)	0.05 (− 0.1 to 0.2)	− 0.1 (− 1.0 to 0.8)
LDL-c (mmol/l)	− 1.6 (− 4.6 to 1.4)	− 6.1 (− 21.6 to 9.3)	− 0.04 (− 0.5 to 0.4)	− 0.04 (− 0.4 to 0.3)	− 1.1 (− 2.8 to 0.6)
FFA (mmol/l)	− 3.5 (− 13.7 to 6.7)	− 32.9 (− 85.0 to 19.2)	− 0.2 (− 1.8 to 1.4)	− 0.2 (− 1.5 to 1.0)	**− 5.7 (− 11.2 to 0.2)**
Triglycerides (mmol/l)	− 0.3 (− 3.5 to 2.8)	7.2 (− 8.8 to 23.1)	**0.5 (0.008 to 0.9)**	0.1 (− 0.3 to 0.5)	− 0.2 (− 2.0 to 1.6)
OSA-related variables					
AHI	**0.8 (0.3 to 1.2)**	2.5 (− 0.03 to 5.1)	**0.1 (0.01 to 0.2)**	**0.1 (0.02 to 0.1)**	0.06 (− 0.3 to 0.4)
sqrtAHI	**3.6 (1.3 to 5.9)**	**13.2 (1.1 to 25.2)**	**0.5 (0.1 to 0.8)**	**0.4 (0.08 to 0.6)**	0.4 (− 1.09 to 2.0)
Average O_2_-dip (%)	− 1.9 (− 7.2 to 3.5)	− 18.0 (− 44.0 to 8.0)	− 0.2 (− 1.0 to 0.6)	− 0.3 (− 0.9 to 0.3)	− 2.3 (− 5.4 to 0.8)

*not corrected

Furthermore, we found that higher IVST was associated with a number of cosevere risk factors, namely higher fasting insulin (*B* = 0.005; 95% CI 0.002 to 0.007), higher HOMA-IR (*B* = 0.1; 95% CI 0.04 to 0.2), lower HDL-c (*B* = − 1.2; 95% CI − 2.2 to 0.1), higher triglyceride levels (*B* = 0.5; 95% CI 0.001 to 0.9), and higher AHI (*B* = 0.1; 95% CI 0.01 to 0.2), independent of BMI z score. After further correcting for age and gender, significance persisted for AHI, HOMA-IR, and fasting insulin levels.

## Discussion

The major findings from our study population of severely obese adolescents are as follows: (1) LVH is highly prevalent and is mostly of the eccentric type; (2) higher LVMI is not significantly associated with anthropometric measures but is independently associated with male gender and higher AHI, the latter implying a pathophysiological relationship between nocturnal hypoxemia and LVH; (3) higher IVST is associated with male gender, lower insulin sensitivity, adverse lipid profile, and higher AHI, independent of BMI z score, which suggests that IVST is associated with an adverse metabolic profile in severely obese adolescents.

Multiple studies describing geometrical changes in obese children have been performed, though they are limited by a high level of intra- and interstudy variability in population characteristics (e.g., a wide range of age, BMI, and ethnicity) and methodology (e.g., different definitions of geometrical aberration), resulting in a reported prevalence of LVH ranging from 10 to 46% [1–6]. Additionally, some studies report that the most common change is eLVH [1, 2], while others report a higher prevalence of cLVR [3] or cLVH [4]. Moreover, data regarding severely obese adolescent patients is scarce, and recent literature is limited to a single study by Ippisch et al. [4]. In our study population of 43 severely obese adolescents aged 14–16 years old (average 15.7 ± 1.0 years), we found that 60.5% suffered from abnormal left ventricular geometry overall, with eLVH in 48.8% and cLVH in 4.6%. Ippisch et al*.* describe a similar prevalence of abnormal left ventricular geometry of 64% in their population of 13–19 year old patients with a mean BMI of 60 ± 9 kg/m^2^, though they used the adult cut-off of the LVMI of 51 g/m^2.7^ [4]. When applying the same cut-off of LVMI in our population, only six patients (14.0%) would suffer from LVH, possibly because the population in the study of Ippisch et al*.* had on average an even higher BMI than our population (15.8 kg/m^2^ difference). We recommend that future studies evaluate more specifically defined populations and determine left ventricular geometry by utilization of LVMI (g/m^2.7^) and RWT, using the pediatric 95^th^ percentile cut-off values of 38.6 g/m^2.7^ and 0.41 cm, respectively, or reporting results using both the pediatric and adult cut-off values [24, 25, 27].

The association between anthropometric measures, in particular BMI and abdominal circumference, with metabolic syndrome is well established [28]. Similarly, several anthropometric variables, including BMI, waist circumference, waist-hip ratio, and waist-length ratios are associated with LVM in adults [29–34]. The pediatric literature relating anthropometric measures with LVM is limited [5]. Daniels et al*.* described a significant association between LVM and body fat percentage [5]. Within our homogeneous study population of severely obese adolescents with a BMI of 44.2 kg/m^2^ (± 5.5), we did not find an association between LVH and any anthropometric variable, such as BMI, neck circumference, and abdominal circumference. Our findings by them self could suggest that in this population at high risk for LVH, additional anthropometric measurements are not valuable to stratify risk. However, the findings by Ippisch et al*.* raise the question whether further increases in BMI are associated with an increased severity of left ventricular hypertrophy [4].

Our hypothesis that higher LVM could be independently associated with an adverse metabolic profile was based on the adult literature that shows an independent association between higher LVM, insulin resistance, and dyslipidemia [35–39]. We did not find any such association with LVM or LVMI. By contrast, we found that IVST is associated with several variables related to comorbidity, namely HOMA-IR, HDL-c, triglyceride levels, and AHI, independent of BMI z score. After further correction for age and gender, associations remain significant for insulin levels, HOMA-IR, and AHI. Previously, multiple studies established that increased IVST is associated with an adverse metabolic profile [40–42], higher blood pressure in young healthy adults [43, 44], and higher all-cause mortality in adults with cardiovascular disease [45, 46]. Our findings support the hypothesis that hyperinsulinemia plays a role in the nonhemodynamic pathogenesis of cardiac hypertrophy, albeit limited to the ventricular septum. Multiple pathogenic mechanisms by which insulin resistance contributes to altered left ventricular geometry are proposed in the literature, including increased fatty acid oxidation with accumulation of cardiotoxic metabolic intermediates and direct binding of insulin to insulin-like growth factor 1 receptors, resulting in cardiomyocyte proliferation [47–51].

We did not find an association between blood pressure and any parameter of left ventricular geometry (LVMI, LVM, IVST, LPWT, or LVEDD), possibly because the levels of systolic and diastolic blood pressure in our population were relatively normal, with z scores of 0.7 (± 1.0) and 0.3 (± 0.8), respectively. These findings are in conjunction with the observations of the Bogalusa heart study, which found that increased left ventricular mass in obese children and young adults preceded the development of increases in systolic and diastolic blood pressure [14]. This further emphasizes the importance of considering obesity as an independent modifiable risk factor for cardiac geometrical changes.

OSA is a well-established independent risk factor for LVH in adults, both through hemodynamic and nonhemodynamic mechanisms [52]. Cloward et al*.* demonstrated that treatment of OSA in adults resulted in a reduction in interventricular septal thickness [53]. Amin et al*.* found that in children and adolescents, an AHI > 10 was associated with a significantly higher risk of LVH when compared to primary snorers (odds ratio 11.2, 95% confidence interval 1.9–64) [54]. Chan et al*.* evaluated children between 6 and 13 years old with or without OSA and found that higher AHI values were associated with increased IVST and RWT [55]. Furthermore, they found that improvement of OSA resulted in improvement of these variables [55]. Recently, Hanlon et al*.* compared the prevalence of LVH in 61 subjects with or without OSA, with a mean age of 13.1 years and a median BMI of 36.3 kg/m^2^ [6]. They found that OSA was an independent risk factor of LVH and that the risk of LVH increased with the severity of OSA. In our study, we found that square-root-transformed AHIs were associated with LVMI, LVM, IVST, and LVPWT, independent of BMI z score. Regression coefficients remained significant, also after further correction for age and gender. Importantly, OSA is highly prevalent in severely obese adolescents, with 86.9% of our patients having an AHI ≥ 1 and 39.5% having an AHI ≥ 5, corresponding to mild and moderate-severe OSA, respectively [56]. Our findings support the notion that OSA is an important pathogenic factor in obesity-related LVH and indicate the importance of early recognition and treatment of this serious comorbidity.

The main strengths of our study are a high level of methodological standardization and extensive evaluation across several relevant clinical domains. Our study population consists of well-defined prospectively recruited severely obese adolescents aged 14–16 years, allowing adequate characterization of this specific population. We were able to evaluate multiple hypotheses related to the determinants of LVH in severely obese adolescents, owing to the extensive data we have on the overall condition of our study population, including hemodynamic factors, metabolic aberrations, and polysomnographic characteristics.

Our study has a number of limitations. A significant number of patients did not receive cardiac ultrasound due to logistic reasons, resulting in a limited sample size. This may have hampered the statistical significance of certain findings and did not allow us to investigate all risk factors in one model, i.e., correcting for the influence of these factors on each other. Moreover, our population concerns mainly females, and therefore, the applicability of these findings to male severely obese adolescents cannot be guaranteed.

We conclude that LVH, especially eLVH, occurs in the majority of severely obese adolescents and that adverse geometrical changes, in particular increased IVST, are independently associated with multiple commonly occurring nonhemodynamic risk factors, including OSA, dyslipidemia, and insulin resistance. We recommend low threshold of suspicion of these risk factors and proactive treatment, in order to potentially improve cardiovascular prognosis. Future research should focus on the effect of reversal of these risk factors on cardiac geometry.

## Abbreviations

AHI: Apnea hypopnea index
BMI: Body mass index
cLVH: Concentric left ventricular hypertrophy
cLVR: Concentric left ventricular remodeling
eLVH: Eccentric left ventricular hypertrophy
DBP: Diastolic blood pressure
HOMA-IR: Homeostatic model assessment of insulin resistance
IVST: Interventricular septal thickness
LVEDD: Left ventricular end-diastolic dimension
LVESD: Left ventricular end-systolic dimension
LVH: Left ventricular hypertrophy
LVM: Left ventricular mass
LVMI: Left ventricular mass index
LVPWT: Left ventricular posterior wall thickness
OSA: Obstructive sleep apnea syndrome
RWT: Relative wall thickness
SBP: Systolic blood pressure
